# Comparing Electromyographic Muscle Activities and Kinematics During Sit-to-Stand Transitions in Patients with Adult Spinal Deformity Versus Healthy Controls

**DOI:** 10.3390/jcm14072514

**Published:** 2025-04-07

**Authors:** Yukako Hayamizu, Tetsuyuki Nagafusa, Kumi Sasaki, Masaaki Nagashima, Katsuya Yamauchi, Tomohiko Hasegawa, Go Yoshida, Tomohiro Banno, Hideyuki Arima, Shin Oe, Tomohiro Yamada, Yukihiro Matsuyama, Yu Yamato

**Affiliations:** 1 Department of Rehabilitation Medicine, Hamamatsu University School of Medicine, Hamamatsu 433-3192, Japan; yukako.h0123@gmail.com (Y.H.);; 2 Department of Rehabilitation Medicine, Tojun Hospital, Jiseikai, Social Medical Corporation, Tokyo 121-0075, Japan; 3 Department of Orthopaedic Surgery, Hamamatsu University School of Medicine, Hamamatsu 433-3192, Japan

**Keywords:** electromyography, kinematics, activities of daily living, spine, lower extremity

## Abstract

**Background/Objectives:** Adult spinal deformity (ASD) affects sit-to-stand (STS) transitions due to abnormal spinal alignment, influencing muscle function. This study investigated lower-extremity electromyographic activity and kinematic parameters during STS transitions in ASD patients. **Methods:** A cross-sectional study was conducted with ASD patients scheduled for corrective surgery. The STS task was divided into three phases, and electromyographic activity, temporal parameters, and joint kinematics were compared between ASD patients and controls. Surface electromyography measured muscle activity, and a high-speed camera recorded phase durations and joint movements. **Results:** Compared to 17 controls, 17 ASD patients exhibited significantly increased %MVIC (ASD, controls, *p*-value) in the biceps femoris during the flexion momentum phase (23.7 ± 26.5, 12.3 ± 8.6, *p* = 0.048) and extension phase (48.6 ± 25.8, 32.8 ± 40.5, *p* = 0.011), and in the soleus during the flexion momentum phase (16.2 ± 7.5, 8.5 ± 2.9, *p* = 0.001). The ASD group also showed greater joint motion and longer phase durations during STS transitions. **Conclusions:** ASD patients display increased lower limb muscle activation, prolonged phase durations, and more joint motion during STS transitions. These findings highlight neuromuscular and biomechanical differences, though whether these are pathological, adaptive, or compensatory remains unclear.

## 1. Introduction

Adult spinal deformity (ASD) is prevalent among individuals aged ≥65 years (32–68%) [1]. The aging process alters all spinal structures, including intervertebral discs, facet joints, vertebral bodies, and the associated musculature and ligaments, resulting in spinal deformities [2]. These structural changes adversely impact patients’ health-related quality of life (HRQOL) and restrict their activities of daily living (ADLs) [3,4].

Sit-to-stand (STS) movements are closely related to HRQOL, and declines in STS performance among older adults may indicate increased dependence, a higher risk of institutionalization, and an elevated likelihood of falls [5,6]. The STS movement is an important functional activity performed approximately 60 times per day [7]. Therefore, analyzing STS movements is crucial for understanding the HRQOL and daily functioning challenges of patients with ASD.

STS transitions depend on multiple factors, including lower-limb muscle strength, proprioception, balance, and accurate postural control [8,9]. Spinal malalignment in ASD leads to compensatory structural adaptations in the hip and knee joints [10,11], contributing to diminished muscle strength, impaired balance, and pain [12,13]. Consequently, it is hypothesized that patients with ASD utilize distinct movement strategies during STS compared to healthy adults. Although prior studies [14,15] have examined spinal alignment and joint movements of the trunk and lower limbs during STS in patients with ASD, none have assessed lower-extremity muscle activity. Research indicates that surface electromyography (sEMG), a non-invasive technique for monitoring real-time muscle activity, is ideal for evaluating the muscle coordination required for STS transitions [16].

This study aimed to use sEMG to investigate the muscle activity and kinematic characteristics of the lower limbs during STS transitions in patients with ASD. The findings may also provide valuable insights for making decisions regarding rehabilitation strategies and surgical treatments for patients with ASD.

## 2. Materials and Methods

Ethics statement: All procedures involving human participants followed the ethical standards of the institutional and/or national research committee and the 1964 Declaration of Helsinki and its later amendments or comparable ethical standards. The study was approved by the Institutional Review Board of Hamamatsu University School of Medicine (IRB approval no., 21-015 and date of approval: 31 March 2021). Informed consent and consent for publication were obtained from the participants.

Study Design: In this cross-sectional study, we recruited patients with ASD scheduled for corrective surgery at Hamamatsu University Hospital between 1 October 2021, and 31 October 2022. Inclusion criteria comprised female sex, age 45–85 years, spinal deformities meeting the International Spine Study Group criteria [17], and ability to stand and ambulate independently without upper extremity support. The exclusion criteria encompassed the absence of muscle weakness or sensory disturbance associated with ASD, neuromuscular disorders, prior spinal or lower extremity joint surgeries, severe systemic diseases, and cognitive impairments preventing comprehension and test completion. Notably, although no randomization method was employed to eliminate selection bias, the selection of subjects was conducted by two researchers to mitigate bias as much as possible. The control group consisted of community-dwelling healthy female older adults recruited from hospital staff or their referrals. Exclusion criteria for the control group comprised a history of ASD, visible spinal deformity, prior spinal or lower-extremity joint surgery, neuromuscular or orthopedic conditions affecting STS performance, chronic back pain impacting ADLs, and trunk tilt during ambulation. Participants meeting the control group’s inclusion criteria were age-matched with those in the ASD group. Demographic data were collected through questionnaire interviews.

### Measurements

Test procedure

**Figure 1:**
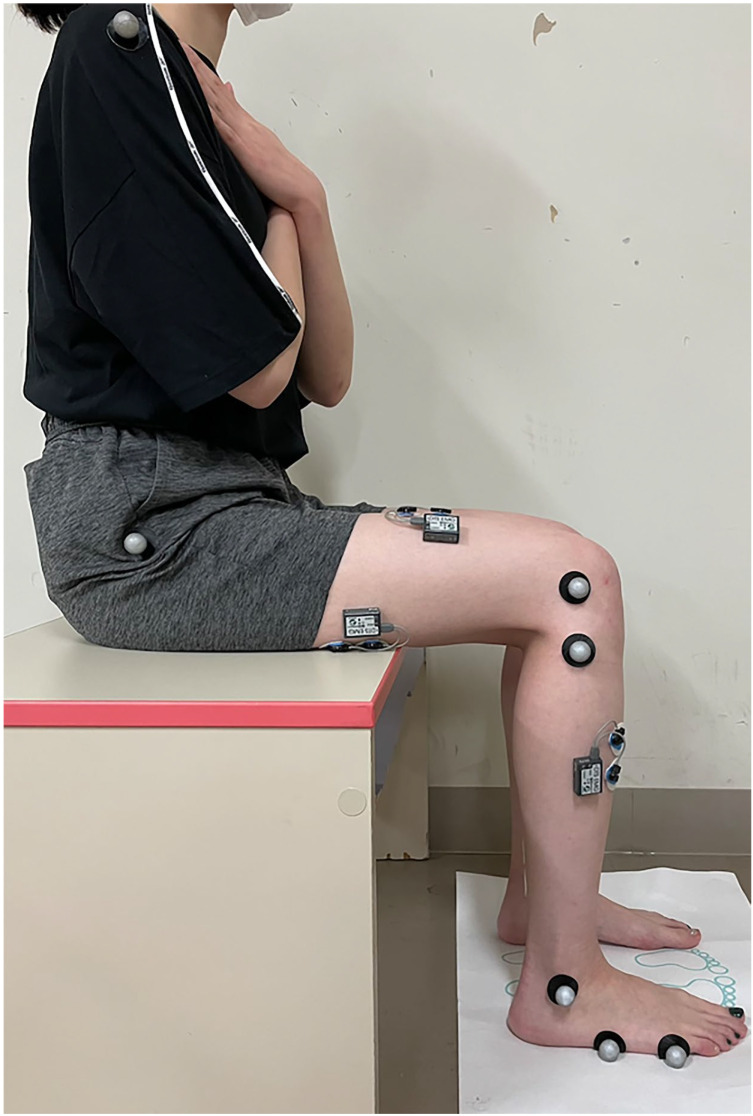
Starting position for sit-to-stand movement measurements. The upper limbs are crossed in front of the chest, with the ankle joint in a neutral position and the knee joint flexed at 90°.

The measurements were conducted by two physical therapists. STS movement was measured from the seated position to standing. Participants were seated on chairs without backrests or armrests, with seat height adjusted to the knee joint cleft. The starting position required participants to sit with arms crossed over the chest, ankles in a neutral position, and knees flexed at 90° [18]. When the individuals who started STS with a “1-2-3 Start” command came to the upright and stable position, they finished STS with a “1-2-3 End” command. Simultaneous sEMG and kinematic recordings were obtained, with each participant performing five trials [19]. Figure 1 illustrates the starting positions for STS movement measurements.

Data acquisition

Percentage maximum voluntary isometric contraction (% MVIC)

%MVIC was derived from sEMG waveforms recorded during both MVIC measurement and the STS test. sEMG was performed using the Telemyo DTS system (Noraxon, Scottsdale, AZ, USA) to assess muscle activity during STS transitions. Monitored muscles included the right rectus femoris (RF), biceps femoris (BF), tibialis anterior (TA), and soleus (Sol). Disposable silver–silver chloride electrodes (Blue Sensor M, Ambu, Baltorpbakken, Denmark) were placed 2 cm apart on each muscle. The system specifications included a common-mode rejection ratio > 100 dB, input impedance > 100 MΩ, a sampling frequency of 1500 Hz, and an EMG measurement bandwidth (bandpass filter) of 10–500 Hz. Before electrode application, the skin was abraded with gel and cleansed with alcohol to minimize impedance. Electrodes were placed according to Surface EMG for Non-Invasive Assessment of Muscles guidelines—RF electrodes were positioned at the midpoint between the anterior superior iliac spine and the patella; BF electrodes at the midpoint between the ischial tuberosity and the lateral femoral condyle; TA electrodes one-third of the way above the line connecting the fibular head and the lateral malleolus; and Sol electrodes two-thirds of the way above the line connecting the medial femoral condyle and the medial malleolus [20]. Following electrode placement, resting muscle activity was recorded to ensure signal fidelity.

**Figure 2:**
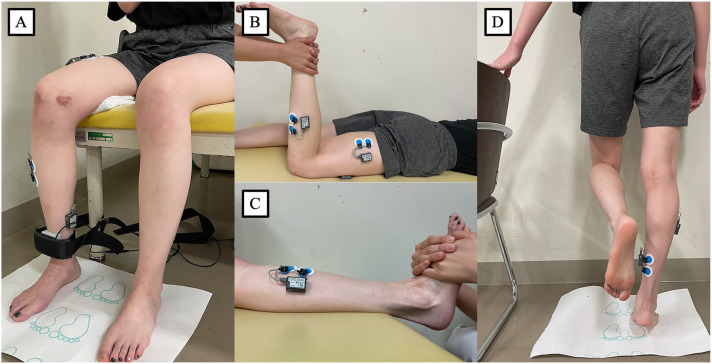
Positions for maximum voluntary isometric contraction measurement. (**A**) Rectus femoris, (**B**) biceps femoris, (**C**) tibialis anterior, (**D**) soleus.

MVIC was determined as the highest contraction force achieved over 6 s against either belt fixation or manual resistance. Each muscle was tested three times, with a one-minute rest interval between trials. MVIC for the four muscles was measured according to Halaki and Ginn’s protocol [21], as follows: (A) RF measured by sitting with the hip flexed at 90°, knee flexed at 90°, arms folded across the chest, and knee extended, while the ankle was secured with a belt; (B) BF measured while lying prone with the knee flexed at 90°, with manual resistance applied at the ankle; (C) TA measured while lying supine with the heel over the bed’s edge, dorsiflexing the ankle from the mid position against resistance applied to the dorsal foot; (D) Sol measured standing on the right leg with the heel off the floor while the left hand grasped a chair for balance. Figure 2A–D illustrates these measurement postures.

Following MVIC measurements, participants performed the STS test. After STS trials, sEMG data were analyzed using MyoMuscle (https://www.noraxon.com/, accessed on 24 March 2025, Noraxon, Scottsdale, AZ, USA). The sEMG waveforms were full-wave rectified and smoothed using a root-mean-square algorithm with a 100 ms interval. The MVIC value was obtained by averaging the sEMG waveforms over 3 s before and after the peak amplitude. The sEMG signals from the middle three of the five STS movements were analyzed, and the %MVIC was calculated as the sEMG amplitude during STS divided by the MVIC value [21].

Kinematic data

Kinematic data included hip, knee, and ankle joint angles and STS movement time. STS transitions were recorded using a Noraxon High-Speed Camera EM-V125N (frame rate—100 fps, resolution—736 × 352 dpi) (Noraxon, Scottsdale, AZ, USA). The camera was positioned on the participant’s right side and synchronized with sEMG recordings. Reflective markers were placed on the right-side acromion, greater trochanter, lateral femoral condyle, fibular head, lateral malleolus, and the fifth metatarsal base and head.

Kinematic data were analyzed using MyoResearch (Noraxon, Scottsdale, AZ, USA). STS movement was divided into three phases based on kinematic characteristics [22]—Phase 1 (flexion momentum phase), from the initiation of the movement to just before the buttocks were lifted off the chair; Phase 2 (momentum-transfer phase), from when the buttocks were lifted off the chair to maximum ankle dorsiflexion; and Phase 3 (extension phase), from just after maximal ankle dorsiflexion to when hip extension ceased. Joint angles for hip flexion, knee flexion, and ankle dorsiflexion were analyzed at the start posture (sitting before STS), between Phases 1 and 2, between Phases 2 and 3, and at the end posture (standing after STS). The duration of each phase and the total STS time were also calculated.

Radiographic measurements

The measurements were conducted by one physical therapist and one physician. For the ASD group, spinal alignment was assessed using whole-spine radiographs in the sagittal and coronal planes while the participant was in an upright position. The spinal alignment parameters measured included the Cobb angle, C7 plumb line-central sacral vertical line, sagittal vertical axis (SVA), lumbar lordosis (LL), thoracic kyphosis (TK), sacral slope (SS), pelvic incidence (PI), and pelvic tilt (PT). These measurements assessed the degree of spinal deformity in the ASD group, but were unrelated to the test procedure.

Statistical analyses: Sample size calculation was based on a previous study [19], with a mean difference in the %MVIC of the RF of 12.7, and standard deviations of 16.5 and 5.6 for the ASD and control groups, respectively, yielding an effect size (d) of 1.03. With a significance level (α) of 0.05 and test power (1 − β) of 0.8, the required sample size was 14.7.

Data normality was assessed using the Shapiro–Wilk test. Due to deviations from normal distribution in most parameters, the Mann–Whitney U test was employed for comparisons between the ASD and control groups. Statistical analyses were conducted using SPSS version 25.0 (IBM, Armonk, NY, USA), with a significance level set at *p* < 0.05.

## 3. Results

**Figure 3:**
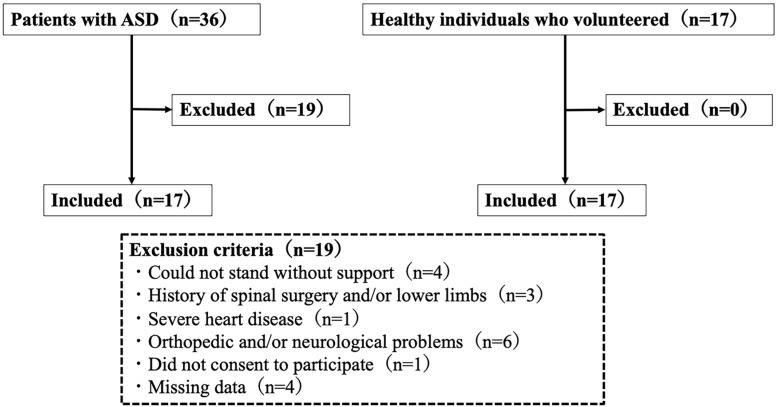
Flow chart of the participant selection process. ASD, adult spinal deformity.

This study included patients with adult spinal deformity (ASD) and healthy volunteers as participants. A total of 36 ASD patients were initially considered, but 19 were excluded based on specific criteria. The exclusion criteria included inability to stand without support (*n* = 4), a history of spinal and/or lower limb surgery (*n* = 3), severe heart disease (*n* = 1), orthopedic and/or neurological disorders (*n* = 6), refusal to participate (*n* = 1), and missing data (*n* = 4). As a result, 17 ASD patients were included in the study. In contrast, 17 healthy individuals volunteered to participate, with no exclusions. Consequently, the final study cohort comprised 34 participants, including 17 ASD patients and 17 healthy controls (Figure 3).

The demographic characteristics of the participants are summarized in Table 1. There were no significant differences between the ASD and control groups in terms of age (ASD—72.7 ± 6.8 vs. Control—71.9 ± 5.8, *p* = 0.569), body mass index (ASD—22.2 ± 4.4 vs. Control—22.7 ± 3.3, *p* = 0.459), or knee extension strength (right knee extension strength, ASD—18.2 ± 5.5 vs. Control—22.1 ± 7.4, *p* = 0.109; left knee extension strength, ASD:—7.3 ± 6.5 vs. Control—21.2 ± 7.2, *p* = 0.109). Spinal alignment parameters (SVA, PT, and PI-LL) assessed using the Scoliosis Research Society–Schwab classification [17] indicated severe sagittal malalignment in the ASD group (SVA—114.3°, PT—33.8°, and PI-LL—30.4°).

sEMG: The %MVIC was significantly higher in the ASD group than in the control group for the BF in Phase 1 (ASD—23.7 ± 26.5 vs. Control—12.3 ± 8.6, *p* < 0.05), BF in Phase 3 (ASD—48.6 ± 25.8 vs. Control—32.8 ± 40.5, *p* < 0.05), and Sol in Phase 1 (ASD—16.2 ± 7.5 vs. Control—8.5 ± 2.9, *p* < 0.05). No significant differences were observed for the RF and TA across all phases (Table 2).

### Kinematics

Joint angles

The joint angles during STS movements were significantly greater in the ASD than in the control group at several points, as follows: the start posture—hip flexion (ASD—78.6 ± 5.8° vs. Control—72.2 ± 5.3°, *p* < 0.05) and knee flexion (ASD—93.2 ± 6.4° vs. Control—88.4 ± 5.8°, *p* < 0.05); between Phases 1 and 2—hip flexion (ASD—111.3 ± 8.5° vs. Control—95.5 ± 10.0°, *p* < 0.05) and knee flexion (ASD—88.2 ± 7.0° vs. Control—82.8 ± 6.0°, *p* < 0.05); between Phases 2 and 3—hip flexion (ASD—103.5 ± 9.9° vs. Control—86.7 ± 10.1°, *p* < 0.05) and knee flexion (ASD—81.9 ± 8.2° vs. Control—76.2 ± 6.0°, *p* < 0.05); the end posture—hip flexion (ASD—18.4 ± 11.9° vs. Control—−1.0 ± 4.9°, *p* < 0.05), knee flexion (ASD—21.9 ± 8.7° vs. Control—7.8 ± 5.0°, *p* < 0.05), and ankle dorsiflexion (ASD—9.0 ± 5.9° vs. Control—4.2 ± 6.7°, *p* < 0.05) (Table 3).

Phase times and total STS time

The duration of each phase and the total STS time were significantly longer in the ASD than in the control group, as follows: Phase 1 (ASD—0.79 ± 0.22 s vs. Control—0.62 ± 0.09 s, *p* < 0.05); Phase 3 (ASD—1.28 ± 0.42 s vs. Control—0.99 ± 0.25 s, *p* < 0.05); and total STS time (ASD—2.29 ± 0.67 s vs. Control—1.80 ± 0.33 s, *p* < 0.05). No significant differences were found in Phase 2 time (ASD—0.22 ± 0.13 s vs. Control—0.19 ± 0.12 s, *p* = 0.326) (Table 4).

## 4. Discussion

This study has investigated muscle activity and kinematic characteristics during STS transitions in patients with ASD. To our knowledge, this is the first report to concurrently assess muscle activity and kinematic indices during STS using sEMG. The findings of this study indicate that muscle activity and joint kinematics during STS movements differ between patients with ASD and healthy adults. These findings provide insights into the physical function of patients with ASD, and may help develop strategies to prevent decline in STS transition ability.

Although no significant difference was observed in MVIC knee extensors between the ASD and control groups, the ASD group exhibited a higher %MVIC of the BF and Sol during STS transitions. In Phase 1, which involves shifting the body’s center of gravity from the seat to the feet, increased trunk forward tilt and hip flexion were required. This phase relies on the gluteus maximus, BF long head, adductor magnus, and plantar flexors for vertical and forward accelerations [23]. Greater trunk forward lean is associated with increased BF muscle activity, and the Sol is activated alongside hip extensors [24]. The ASD group’s larger hip flexion angle between Phases 1 and 2 suggests an increased %MVIC of BF and Sol during Phase 1 STS movements. Anatomically, the BF, which extends from the ischial tuberosity to the fibular head, may experience increased activity due to greater pelvic anterior tilt, which lengthens the muscle [25]. In addition, individuals with ASD generally exhibit greater pelvic tilt, and it is possible that the biceps femoris muscle could be shortened, which might influence the increase in %MVIC of the biceps femoris. However, since pelvic tilt during the start posture (sitting before STS) was not measured in this study, it should have been measured.

**Figure 4:**
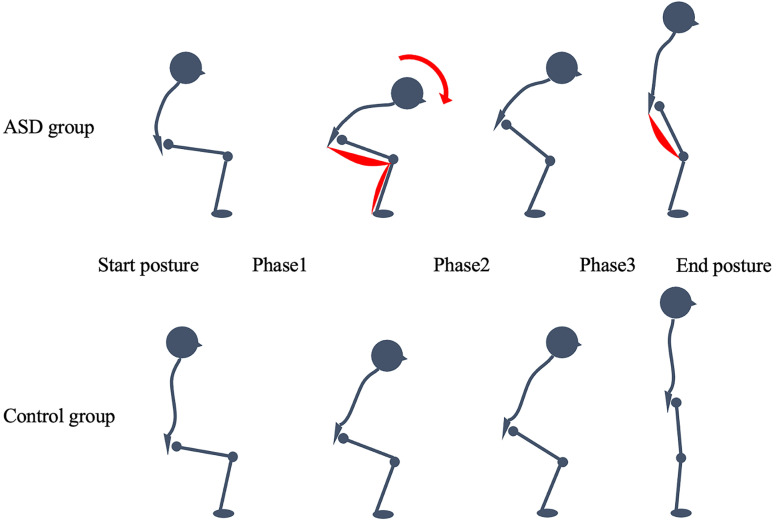
Example of movement observed in patients with ASD and healthy controls. Phase 1: Flexion momentum. Phase 2: Momentum transfer. Phase 3: Extension. ASD, adult spinal deformity.

In Phase 3, the ASD group demonstrated an increased %MVIC of the BF compared to the control group. The momentum generated in Phase 1 propels the body forward, with continued anterior and upward movement of the center of mass in Phase 2. In Phase 3, the momentum from the upper body is transferred to the entire body, reducing the need for lower-extremity muscle force. However, the slower initial movement in Phase 1 results in reduced momentum, requiring greater effort from the lower limb muscles [26]. The significantly longer STS movement time in Phase 1 for the ASD group suggests that Phase 3 relies more on lower-extremity extensor muscles, as evidenced by the increased %MVIC of BF (Figure 4).

Interestingly, no significant difference was observed in the %MVIC of BF and Sol in Phase 2. As the center of mass shifts forward from Phase 1 to Phase 2, trunk movement, hip flexion, and ankle dorsiflexion continue. We have speculated that BF and Sol activity would increase, leading to a higher %MVIC; however, our findings do not align with this assumption. The reason for this discrepancy remains unclear, but may be attributed to high data variability, leading to large differences in average values.

Furthermore, STS movement times were longer in the ASD group compared to the control group in Phase 1, Phase 3, and total time. STS is a complex movement involving lower-limb strength, sensory input, balance, and postural control [8,9]. In this study, we did not investigate fatigue, discomfort or balance impairments, but it has been reported that frail older individuals and those with impaired balance typically exhibit longer STS times and increased trunk forward tilt for stabilization [26,27]. Consequently, reduced balance and altered neuromuscular control due to spinal deformities and compensatory mechanisms likely affected our participants [12,13]. Additionally, patients with ASD may show reduced hip and lumbar spine movement velocities to mitigate pain [28]. These findings, as well as the prevalence of pain among patients with ASD, may have contributed to slower movement speeds.

Patients with ASD and healthy adults exhibited distinct differences in muscle activity and joint movements during STS. However, whether these differences were caused by abnormal spinal alignment or functional limitations of ASD remains unclear. As ASD is a progressive condition primarily associated with aging [2], even individuals who are currently independent in the STS task may experience impaired function with disease progression [29]. An impaired STS ability is associated with increased dependence, institutionalization, fall risk, and prolonged hospital admissions [6,7], potentially predicting adverse outcomes for such patients. However, this study did not investigate the functional relevance of patient independence or previous falls. Therefore, there were significant differences in %MVIC and joint movement between ASD and healthy individuals, but it is unclear to what extent these differences are functionally relevant for patient independence and fall risk. Understanding the differences in the characteristics of ASD and healthy individuals’ %MVIC and joint movements is essential to maintaining physical function and independence. Future research should focus on clarifying how the differences in STS ability characteristics between individuals with ASD and healthy controls contribute to functional impairments, and on developing rehabilitation strategies and treatment methods to maintain and improve STS capability in patients with ASD.

This study has some limitations. First, this study included only 17 female patients with ASD and 17 healthy controls, resulting in a small sample size, which limits the applicability to male patients or those with different spinal deformity patterns. Second, we did not evaluate the muscle activity of the erector spinae and gluteus maximus, which are crucial for STS movements. Third, only right-side muscle activity was assessed. Therefore, the results may be influenced by leg dominance, habitual exercise patterns, or existing muscle weakness. Abnormal spinal alignment may influence erector spinae and lower-extremity muscle activity [12,13], and lumbar scoliosis can lead to asymmetric sEMG activity [30]. Furthermore, the results were not evaluated based on the severity of ASD. Therefore, these factors should be considered when interpreting the results. Fourth, only spinal alignment in the standing position was assessed. Finally, this study does not measure pain levels, balance confidence, or functional independence, making it unclear whether the observed biomechanical changes translate to real-world disability.

## 5. Conclusions

Muscle activity and joint kinematics during STS movements differ between patients with ASD and healthy adults. These findings provide insights into the physical function of patients with ASD, and may help develop strategies to prevent decline in STS transition ability. Further research with larger cohorts is needed to identify optimal physical therapy programs and to assess the impact of ASD on HRQOL.

## Figures and Tables

**Table 1:** Participants’ demographic data.

	ASD Group ^(a), (b)^	Control Group ^(b)^	Z ^(c)^	*p*
	(*n* = 17)	(*n* = 17)		
Age (years)	72.7 ± 6.8	71.9 ± 5.8	−0.570	0.569
Height (cm)	148.3 ± 5.2	151.8 ± 5.8	−1.639	0.101
Weight (kg)	48.9 ± 10.1	52.2 ± 8.0	−1.172	0.241
BMI (kg/m^2^)	22.2 ± 4.4	22.7 ± 3.3	−0.741	0.459
Right knee extension strength (kgf)	18.2 ± 5.5	22.1 ± 7.4	−1.602	0.109
Left knee extension strength (kgf)	17.3 ± 6.5	21.2 ± 7.2	−1.636	0.102
Cobb angle (°)	36.5 ± 19.1	N/A	N/A	N/A
C7PL-CSVL (mm)	44.6 ± 37.5	N/A	N/A	N/A
SVA (mm)	114.3 ± 67.1	N/A	N/A	N/A
LL (°)	25.3 ± 13.6	N/A	N/A	N/A
TK (°)	35.2 ± 15.9	N/A	N/A	N/A
SS (°)	21.9 ± 8.6	N/A	N/A	N/A
PI (°)	55.7 ± 10.8	N/A	N/A	N/A
PT (°)	33.8 ± 9.8	N/A	N/A	N/A
PI-LL (°)	30.4 ± 15.0	N/A	N/A	N/A

^(a)^ ASD, adult spinal deformity; BMI, body mass index; C7PL-CSVL, C7 plumb line-central sacral vertical line; SVA, sagittal vertical axis; LL, lumbar lordosis; TK, thoracic kyphosis; SS, sacral slope; PI, pelvic incidence; PT, pelvic tilt. ^(b)^ Values are presented as means ± standard deviations. ^(c)^ Z means test statistic. N/A means not applicable.

**Table 2:** Muscle activities during sit-to-stand (%MVIC).

Muscle	Period	ASD Group ^(a), (b)^	Control Group ^(b)^	Z ^(c)^	*p*
		(*n* = 17)	(*n* = 17)		
Rectus femoris (%)	Phase 1	37.8 ± 16.2	33.3 ± 17.2	−1.223	0.221
	Phase 2	65.9 ± 26.1	64.3 ± 35.2	−0.482	0.630
	Phase 3	54.4 ± 25.3	51.8 ± 27.8	−0.465	0.642
Biceps femoris (%)	Phase 1	23.7 ± 26.5	12.3 ± 8.6	−1.981	0.048 ^(d)^
	Phase 2	84.5 ± 107.3	46.0 ± 54.2	−1.567	0.117
	Phase 3	48.6 ± 25.8	32.8 ± 40.5	−2.532	0.011 ^(d)^
Tibialis anterior (%)	Phase 1	47.5 ± 36.2	36.6 ± 19.1	−0.741	0.459
	Phase 2	47.4 ± 29.0	46.8 ± 31.6	−0.189	0.850
	Phase 3	26.1 ± 17.3	22.2 ± 16.9	−0.500	0.617
Soleus (%)	Phase 1	16.2 ± 7.5	8.5 ± 2.9	−3.393	0.001 ^(d)^
	Phase 2	32.9 ± 23.5	26.1 ± 12.7	−0.448	0.654
	Phase 3	32.0 ± 15.9	30.6 ± 11.0	−0.052	0.959

^(a)^ ASD, adult spinal deformity; MVIC, maximum voluntary isometric contraction. ^(b)^ Values are presented as means ± standard deviations. ^(c)^ Z means test statistic. ^(d)^ Significant difference between the ASD and control groups (*p* < 0.05, Mann–Whitney U test).

**Table 3:** Joint angles during the sit-to-stand test.

	ASD Group ^(a), (b)^	Control Group ^(b)^	Z ^(c)^	*p*
	(*n* = 17)	(*n* = 17)		
Start posture (sitting)				
Hip flexion (°)	78.6 ± 5.8	72.2 ± 5.3	−2.980	0.003 ^(d)^
Knee flexion (°)	93.2 ± 6.4	88.4 ± 5.8	−2.068	0.039 ^(d)^
Ankle dorsiflexion (°)	14.2 ± 7.7	12.0 ± 8.3	−0.913	0.361
Between Phases 1 and 2				
Hip flexion (°)	111.3 ± 8.5	95.5 ± 10.0	−3.772	0.000 ^(d)^
Knee flexion (°)	88.2 ± 7.0	82.8 ± 6.0	−2.292	0.022 ^(d)^
Ankle dorsiflexion (°)	20.6 ± 8.8	16.8 ± 7.5	−1.241	0.215
Between Phases 2 and 3				
Hip flexion (°)	103.5 ± 9.9	86.7 ± 10.1	−3.790	0.000 ^(d)^
Knee flexion (°)	81.9 ± 8.2	76.2 ± 6.0	−2.053	0.040 ^(d)^
Ankle dorsiflexion (°)	23.6 ± 9.1	20.1 ± 8.5	−1.034	0.301
End posture (standing)				
Hip flexion (°)	18.4 ± 11.9	−1.0 ± 4.9	−4.567	0.000 ^(d)^
Knee flexion (°)	21.9 ± 8.7	7.8 ± 5.0	−3.878	0.000 ^(d)^
Ankle dorsiflexion (°)	9.0 ± 5.9	4.2 ± 6.7	−2.171	0.030 ^(d)^

^(a)^ ASD, adult spinal deformity. ^(b)^ Values are presented as means ± standard deviations. ^(c)^ Z means test statistic. ^(d)^ Significant difference between the ASD and control groups (*p* < 0.05, Mann–Whitney U test).

**Table 4:** Duration of phase periods and the total time of the sit-to-stand movement.

	ASD Group ^(a), (b)^	Control Group ^(b)^	Z ^(c)^	*p*
	(*n* = 17)	(*n* = 17)		
Phase 1: Flexion momentum (s)	0.79 ± 0.22	0.62 ± 0.09	−2.290	0.022 ^(d)^
Phase 2: Momentum transfer (s)	0.22 ± 0.13	0.19 ± 0.12	−0.982	0.326
Phase 3: Extension (s)	1.28 ± 0.42	0.99 ± 0.25	−2.222	0.026 ^(d)^
Total (s)	2.29 ± 0.67	1.80 ± 0.33	−2.256	0.024 ^(d)^

^(a)^ ASD, adult spinal deformity. ^(b)^ Values are presented as means ± standard deviations. ^(c)^ Z means test statistic. ^(d)^ Significant difference between the ASD and control groups (*p* < 0.05, Mann–Whitney U test).

## Data Availability

All data generated or analyzed in this study are included in the published article.
